# Sex influences whether hippocampal volumes mediate the relationship between depression and cognition in older adults without dementia: A UK Biobank study

**DOI:** 10.1007/s11682-024-00930-6

**Published:** 2024-10-11

**Authors:** Nancy E. Ortega, Vahan Aslanyan, Judy Pa

**Affiliations:** 1 Alzheimer’s Disease Cooperative Study (ADCS), Department of Neurosciences, University of California, San Diego, La Jolla, CA USA; 2 Department of Population and Public Health Sciences, Keck School of Medicine, University of Southern California, Los Angeles, CA USA

**Keywords:** Sex differences, Hippocampus, Cognition, Depression, MRI

## Abstract

Depression is a modifiable risk factor for dementia; however, it remains unclear whether there are sex differences in how depression affects dementia risk. To better understand sex-specific differences in how depression confers risk of dementia, the link between depression, hippocampal volumes, and cognition was evaluated in a sample of older adults without dementia from the UK Biobank cohort. A total of 18,220 participants (women *n* = 9,474; men *n* = 8,746) were selected based on completion of the Patient Health Questionnaire (PHQ-9), structural MRI, and cognitive assessments. Causal mediation analyses were used to evaluate if the relationship between depression and cognition is mediated by the hippocampus differently by sex. Women reported greater depression severity than men. Hippocampal volumes were found to mediate the relationship between depression severity and fluid intelligence only in women. Upon categorization of the depression symptoms as either cognitive/affective or somatic, the mediation effect of the hippocampus was seen for both cognitive/affective and somatic symptom severity in women for fluid intelligence. These results offer insight into the sex-specific pathways underlying the relationship between depression, hippocampal volumes, and cognition in older adults without dementia with a focus on the type of depression symptoms. This knowledge could aid in the development of sex-focused dementia prevention strategies and treatments.

**Supplementary Information:**

The online version contains supplementary material available at 10.1007/s11682-024-00930-6.

Depression is a common geriatric psychiatric disorder, with prevalence in older adults estimated as high as 31.74% (Zenebe et al., 2021). The effects of depression go beyond personal suffering and disruption of social relationships; it is a debilitating disease that causes functional disability, worsens health outcomes, and leads to mortality via suicide or by increasing an individual’s vulnerability to disease (Alexopoulos, 2005; Péquignot et al., 2019). Depression has been recognized as a modifiable risk factor for dementia, with studies attributing a two-fold increase in the risk of all-cause dementia to late-life depression (Byers & Yaffe, 2011; Livingston et al., 2020). Assuming that depression increases Alzheimer’s disease (AD) risk, this would suggest that 5–10% of AD cases could be prevented or their progression delayed if depression was treated (Norton et al., 2014).

Studies on older adults without dementia report that depression is associated with hallmark features of AD, including cognitive impairment, hippocampal volume loss, and protein misfolding and aggregation (i.e., amyloid beta and tau) (Babulal et al., 2020; Butters et al., 2004; Nascimento et al., 2015; Twait et al., 2024; Videbech, 2004). Evidence for neural pathways by which depression influences cognition, and subsequently AD risk, is sparse. A possible mechanism by which depression could confer risk of AD is via neurotoxicity within the hippocampus caused by depression-associated pathology (Dafsari & Jessen, 2020). Neurotoxic pathology in depressed patients that can result in smaller hippocampal volumes include: altered stress responses, heightened inflammatory responses, and/or decreased circulating neurotrophic factors (Nestler et al., 2002; Sheline et al., 1996). Reduced hippocampal volumes have been linked to cognitive decline in late-life, even when AD pathology is accounted for (Dawe et al., 2020). Research suggests the negative effects of depression on cognition, particularly within the executive function domain, can be ameliorated with antidepressant treatments (Colwell et al., 2022). Together these findings suggest that evaluating the relationship between depression, hippocampal volumes, and cognition provides a pathway by which researchers can investigate how depression increases risk of AD.

A notable feature that is shared between depression and AD that must be considered when investigating causal pathways are sex differences, with women showing a greater lifetime risk of depression and AD relative to men (Dal Forno et al., 2005). Despite sex and gender being recognized as crucial factors that influence prevalence, incidence, disease progression and treatment of these diseases, it is still not fully understood whether sex differences in depression contribute to the sex differences that are seen in AD risk. Studies have shown that men and women differ in the types of depression symptoms endorsed, with women significantly reporting more fatigue, sleep, and psychomotor deficits compared to men who report symptoms like insomnia and agitation (Cavanagh et al., 2017; Khan et al., 2002). It is possible that different biological mechanisms in women and men could result in sex-specific disease-related changes, that would in turn provide valuable information that can be used to tailor preventative interventions. Evaluating the relationship between types of depression symptoms (i.e., cognitive/affective and somatic) and cognition could help identify which individuals are more vulnerable to the effects of depression on their risk for AD.

The objective of this project was to examine whether there are sex differences in how depression confers risk of AD by evaluating the relationship between depression, cognition, and hippocampal volumes. A sub-sample of 18,220 older adults without dementia from the UK Biobank cohort were used to investigate if hippocampal volumes mediate the relationship between depression and cognition differently by sex. An exploratory analysis by depression symptom type (cognitive/affective and somatic) was conducted. It was hypothesized: 1. Women would have greater depression severity; 2. Smaller hippocampal volumes would mediate the relationship between greater depression severity and lower cognitive performance in women more so than in men.

## Methods

### Study cohort: UK Biobank

The UK Biobank is one of the largest and most detailed biomedical datasets containing longitudinal data on 500,000 community-dwelling individuals from the United Kingdom (Sudlow et al., 2015). For more than a decade, assessments evaluating participant demographics, cognitive abilities, lifestyles, mental and physical health were administered across different timepoints in person and online. Brain imaging, such as structural MRI, was acquired on a subset of participants at the third and fourth repeat in-person visits.

### Participant selection

Participants were selected if they had completed the Patient Health Questionnaire (PHQ-9), one of the eight cognitive tests administered at the third in-person visit, and a baseline structural MRI at the third in-person visit (Figure S1). Only individuals 60 years of age or older were included in this study to minimize any effects that could be attributed to menopause. Participants who reported a history of dementia, Alzheimer’s disease or cognitive impairment at the time the data was downloaded were excluded from the present study. All analyses were completed using UK Biobank data downloaded on the 21st of July 2021.

### Depression measures

Depression severity was accessed using data from the self-reported Patient Health Questionnaire (PHQ-9) that was collected as a part of the UK Biobank’s Mental Health Web-based Questionnaire. Previously enrolled UK Biobank participants were invited via email and annual newsletters to complete the questionnaire via their personal devices. The PHQ-9 is a 9-item version of the complete PHQ which indicates presence/absence and severity of current depression. Each item can be scored from 0 (not at all) to 3 (nearly every day), thus the depression severity score can range from 0 to 27. Following research by de Jonge et al. (2007), PHQ-9 items were classified as either a cognitive/affective or somatic symptom. Severity scores for cognitive/affective symptoms were created by summing scores on five PHQ-9 items: lack of interest/pleasure, depressed mood, feelings of inadequacy, concentration problems, and suicidal ideation. Severity scores for somatic symptoms were computed by summing the scores on four PHQ-9 items: sleep problems, low energy, appetite problems, and psychomotor agitation/impairment.

### Cognitive measures

The cognitive tasks were administered on a touchscreen at the assessment center. There were multiple tasks that were administered throughout the visit to cover a broad range of cognitive domains (see Table 1 for a detailed breakdown of the tasks). Distributions for every cognitive test were examined and transformed if the distribution was not normal. The distribution for the Pairs Matching task was the only task that was transformed using log(x + 1) due to it being right skewed.

**Table 1:** UK Biobank Cognitive Tests

Test	Cognitive domain	Task description	Outcome measured	Sample size (% female)	PHQ-9 score > 4 sample (% female)^a^
Fluid Intelligence	Verbal and numerical reasoning	Subjects had 2 min to answer a series of questions aimed to access the capacity to solve problems using logic and reasoning skills, independent of acquired knowledge	Number of questions answered correctly	*N* = 17,881 (52.0%)	*N* = 2,766 (59.5%)
Numeric Memory	Working memory	A backward digit span test	Maximum number of digits remembered correctly	*N* = 12,861 (52.6%)	Sensitivity analyses not applicable
Paired Associate Learning	Verbal declarative memory	Subjects were shown 12 pairs of words (for 30 s), after a break in which they completed a different task, they were presented the first 10 words from the pairs and asked to choose the matching second word from a list of 4 words	Number of questions answered correctly	*N* = 12,634 (52.7%)	*N* = 2,018 (59.9%)
Pairs Matching	Visual declarative memory	Subjects were asked to memorize the position of as many matching pairs as possible, the cards were then turned face down and subjects were then asked to select as many pairs as possible in the fewest tries. This was done for multiple rounds, with each round having more pairs of cards than the next	Number of errors made on the second trial (12 cards; 6 pairs)	*N* = 18,220 (52.0%)	Sensitivity analyses not applicable
Reaction Time	Processing speed	Go/No-Go test where a subject was shown two cards at a time, if the cards were identical the subject had to press a button-box as quickly as possible. There were 12 rounds	Mean reaction time in milliseconds	*N* = 17,553 (51.9%)	Sensitivity analyses not applicable
Symbol Digit Substitution	Processing speed	Subject was presented with a key indicating a symbol and single-digit integer pair, they were then asked to fill in on a separate table which integer belonged with the symbol being presented by following the key they were given. Subjects were instructed to work as quickly as possible	Number of correct symbol-digit matches	*N* = 12,516 (52.7%)	*N* = 1,988 (60.0%)
Tower Rearranging	Executive function	Adapted version of the one-touch Tower of London test. Subjects were shown an image of three towers that had three differently colored hoops through them, they were then asked to indicate how many moves would be required to re-arrange the hoops into another specified position	Number of items answered correctly within 3 min	*N* = 12,386 (52.7%)	Sensitivity analyses not applicable
Trail Making	Executive function	Computerized version of the Halstead-Reitan Trail Making Test; consisted of two trails (Trail #1 & Trial #2); Participants were shown sets of digits and letters in circles that were scattered across the screen, they were then asked to order them sequentially based on the algorithm specific to each trial	Time to complete each trail in deciseconds	*N* = 11,556 (52.7%)	Sensitivity analyses not applicable

The source for this table is https://biobank.ndph.ox.ac.uk/showcase/. ^a^ Sensitivity analyses were conducted on a sub-sample of older adults that scored greater than a 4 on the PHQ-9

### MRI acquisition and processing

This study utilized imaging-derived phenotypes (IDPs) provided by the UK Biobank. These numerical pipeline outputs were generated using an image-processing pipeline that was developed and run by the UK Biobank. This pipeline made use of publicly available image processing tools such as the FMRIB Software Library (FSL, version 5.0.10); tissue-type segmentation was applied using the FMRIB’s Automated Segmentation Tool (FAST) based on the Harvard–Oxford cortical and subcortical atlases and the Diedrichsen cerebellar atlas (Zhang et al., 2001). Detailed documentation for the acquisition parameters and image-processing pipeline can be found within the UK Biobank showcase (https://biobank.ctsu.ox.ac.uk/crystal/crystal/docs/brain_mri.pdf) and elsewhere (Alfaro-Almagro et al., 2018). All participants were scanned using a standard Siemens Skyra 3T scanner running VD13A SP4, with a standard Siemens 32-channel radio-frequency (RF) receive head coil. Briefly, T1-weighted images were obtained using a 3D magnetization-prepared rapid gradient-echo (MPRAGE) sequence: TR = 2,000 ms, TE = 2 ms, 208 sagittal slices, flip angle = 8°, field-of-view = 208 × 256x256 matrix, resolution = 1 × 1x1 mm, length of scan = 5 min. The IDPs used for this study include the hippocampal (left and right hemispheres) volumes and total grey and white matter volumes. To obtain a single volume for the hippocampus, the left and right hemisphere volumes were averaged and converted from mm^3^ to cm^3^ for ease of interpretation of model estimates.

### Statistical analyses

**Fig. 1:**
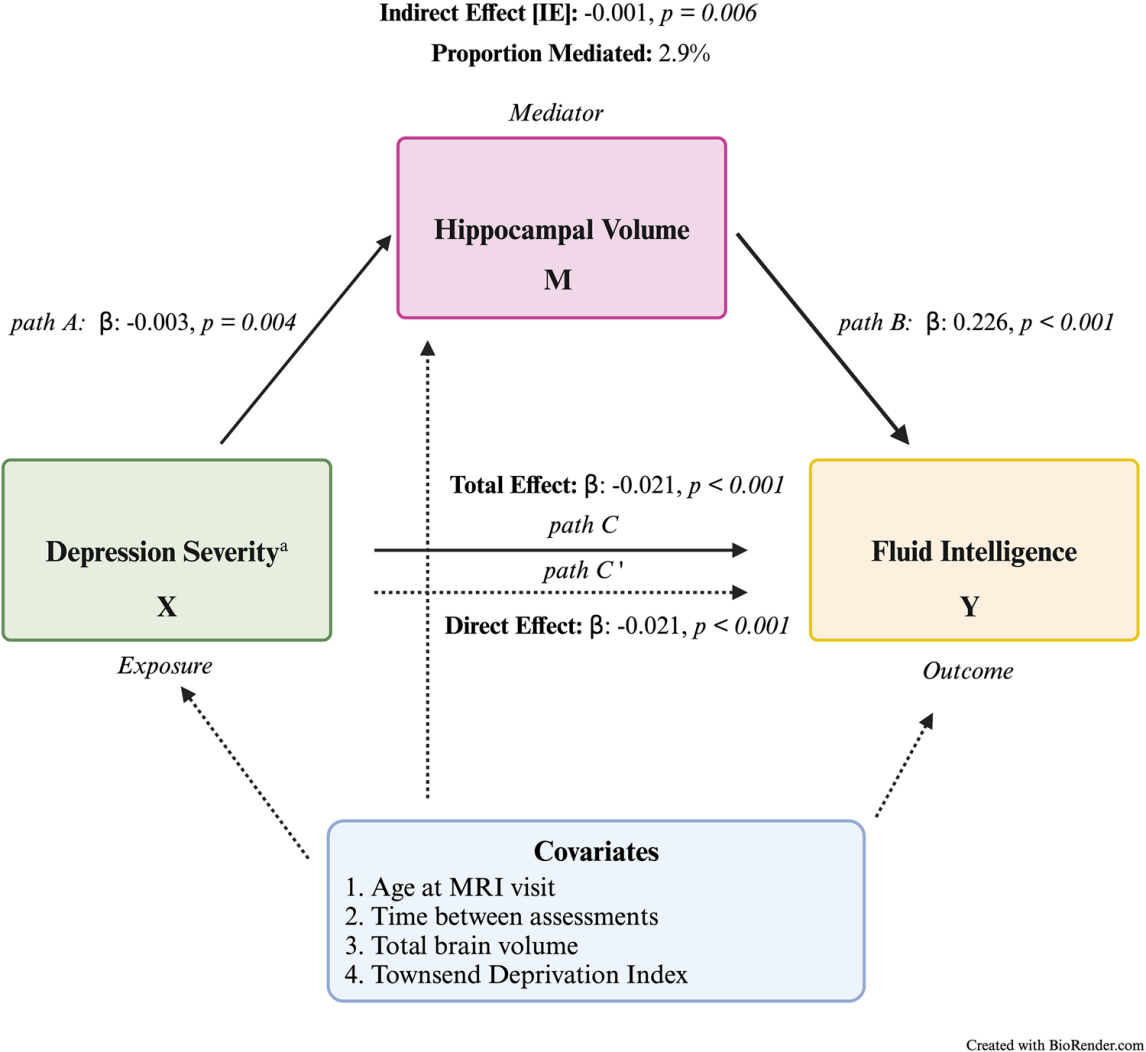
Mediation model for depression severity and fluid intelligence via hippocampal volume in women. *Note*. ^a^Depression severity was measured by the Patient Health Questionnaire (PHQ-9). X: Depression severity total score; M: Hippocampal volume; Y: Cognition measure—Fluid Intelligence score. Adjusted p-values after Benjamini–Hochberg procedure were: Total effect *p* = *0.004*; Direct effect *p* = *0.004*; Indirect effect *p* = *0.019*; Path A *p* = *0.013*; Path B *p* = *0.004*

All statistical analyses were conducted using R Statistical Software (version 4.2.1). Variable distributions were visually inspected. Differences among variables of interest between men and women were evaluated using Student t-tests for continuous variables and Chi-Squared tests for categorical variables. Mediation analyses were conducted using the ‘mediation’ package in R, and by following the Baron and Kenny steps (Baron & Kenny, 1986; Tingley et al., 2014). These steps indicate a mediation analysis is comprised of a set of three regressions: X → Y (path C; total effect), X → M (path A), X + M → Y (path C’; direct effect). If all three steps reached statistical significance the significance of the indirect effect was accessed using bootstrapping procedures. The authors opted to use this stringent criterion for path conditions since it was deemed necessary for the scientific rationale of the study and to reduce the number of tests needed in multiple comparisons analyses. Figure 1 depicts the paths that were evaluated. Depression severity, the exposure variable, had three items that were evaluated: 1) PHQ-9 total score, 2) PHQ-9 cognitive/affective symptom score, 3) PHQ-9 somatic symptom score. Cognitive function, the outcome variable, had eight variables that were evaluated (one outcome variable per cognitive test; Table 1). Hippocampal volume was the only mediator evaluated in this study. Age at the MRI visit, total brain volume, Townsend Deprivation Index (TDI) [a measure of material deprivation], and a computed variable accounting for time between key study variables (i.e., cognitive measurement, MRI scan, and PHQ-9) were used as covariates. A priori sex stratified models were used to evaluate sex differences. Sensitivity analyses were conducted on a sub-sample of older adults that scored greater than a 4 on the PHQ-9 (Table S1). Based on PHQ-9 scoring, a score greater than a 4 indicates mild to severe depression (Kroenke et al., 2001). All results were deemed statistically significant at *p*-value < 0.05. The Benjamini–Hochberg procedure was used to control for false discovery rate.

## Results

### Participant characteristics

The study sample consisted of 18,220 older adults without dementia (52% women, mean age ± standard deviation = 67.9 ± 4.91 years; Table 2), majority of which self-identified as White, were highly educated, and had low material deprivation scores. Men were found to be older and had lower material deprivation scores than women (all *p* < 0.001). The average time between key study variables (i.e., cognitive measurement, MRI scan, and PHQ-9) in years was slightly longer for women (*p* = 0.002).

**Table 2:** Subject Demographics

	Women (*N* = 9,474)	Men (*N* = 8,746)	Overall (*N* = 18,220)	Effect Size	*P-value*
**Demographics**					
Age at First MRI Visit (years)^a^	67.30 ± 4.81	68.50 ± 4.94	67.90 ± 4.91	0.240	< *0.001*
Ethnicity [% White]	98.10%	97.80%	98.00%	< 0.001	*0.957*
Education [% University level or beyond]	57.20%	57.50%	57.30%	0.003	*0.720*
Townsend Deprivation Index^b^	-2.00 ± 2.66	-2.14 ± 2.58	-2.07 ± 2.62	0.052	< *0.001*
Time Between Key Study Variables (years)^c^	1.10 ± 0.59	1.07 ± 0.59	1.09 ± 0.59	0.045	*0.002*
**Depression Measures**					
PHQ-9 Depression Severity Total Score^d^	2.54 ± 3.34	1.94 ± 2.96	2.25 ± 3.18	0.190	< *0.001*
PHQ-9 Diagnosis [% Score < 4]^e^	17.70%	13.10%	15.50%	0.067	< *0.001*
PHQ-9 Cognitive Symptoms Severity Score	0.89 ± 1.82	0.74 ± 1.66	0.82 ± 1.75	0.091	< *0.001*
PHQ-9 Somatic Symptoms Severity Score	1.64 ± 1.86	1.20 ± 1.64	1.43 ± 1.77	0.251	< *0.001*
**MRI Measures**					
Hippocampal Volume (cm^3^)^f^	4.11 ± 0.37	4.39 ± 0.41	4.25 ± 0.41	0.725	< *0.001*
Total Brain Volume (cm^3^)^f^	1,090 ± 85.20	1,210 ± 94.10	1,150 ± 107	1.321	< *0.001*
**Cognitive Measures**					
Fluid Intelligence [*N* = 17,881]^g^	6.62 ± 1.96	6.89 ± 2.06	6.75 ± 2.02	0.134	< *0.001*
Numeric Memory [*N* = 12,861]^h^	6.66 ± 1.23	6.88 ± 1.25	6.76 ± 1.24	0.173	< *0.001*
Paired Associate Learning [*N* = 12,634]^g^	7.27 ± 2.55	6.38 ± 2.56	6.85 ± 2.60	0.345	< *0.001*
Pairs Matching [*N* = 18,220]^i^	1.36 ± 0.62	1.39 ± 0.63	1.37 ± 0.63	0.047	*0.002*
Reaction Time [*N* = 17,553] (milliseconds)^j^	606 ± 89.20	558 ± 88.30	597 ± 89.30	0.213	< *0.001*
Symbol Digit Substitution [*N* = 12,516]^k^	18.30 ± 5.00	18.10 ± 4.86	18.20 ± 4.94	0.053	*0.003*
Tower Rearranging [*N* = 12,386]^l^	9.54 ± 3.15	10.0 ± 3.15	9.76 ± 3.16	0.145	< *0.001*
Trail Making [*N* = 11,556] (deciseconds)^m^	322 ± 147	323 ± 148	322 ± 140	0.006	*0.738*

Sex differences were evaluated by Student’s t-test for continuous variables and with Chi-Square test for categorical variables. Unless otherwise specified assume mean ± standard deviation is presented. Effect size was determined using Cohen’s D for continuous variables and Cramer’s V for categorical variables. ^a^Age range for the sample is 60 to 82 years. ^b^Negative values on the Townsend Deprivation Index indicate low material deprivation. ^c^Time between key study variables (i.e., cognitive measurement, MRI scan, and PHQ-9) was calculated for each subject. ^d^Patient Health Questionnaire (PHQ-9) total score range 0 – 27. ^e^Subjects were categorized into a diagnosis category based on PHQ-9 defined cut-off scores. ^f^These volumes are not normalized for head size. ^g^Outcome: Questions answered correctly. ^h^Outcome: Maximum digits remembered. ^i^Outcome: Number of errors made. ^j^Outcome: Mean time to react. ^k^Outcome: Number of correct matches. ^l^Outcome: Number of items correctly answered. ^m^Outcome: Time to complete trial. All analyses were completed based on UK Biobank data downloaded on the 21st of July 2021

### Sex differences in depression, cognition, and hippocampal volumes

Women endorsed higher scores for all depression severity measures (all *p* < 0.001), with 17.7% of women reporting depression severity scores indicative of mild depression or above. Effect sizes for sex differences in depression severity measures were negligible except for somatic symptoms severity scores (Cohen’s d = 0.251). As seen in Table 2, sex differences in cognitive performance were statistically significant. Men performed better on the Fluid Intelligence, Numeric Memory, Reaction Time, and Tower Rearranging tasks, whereas women performed better on the Paired Associate Learning, Pairs Matching, and Symbol Digit Substitution. No differences were found for the Trail Making task (*p* = 0.738). Men had bigger hippocampal volumes (*p* < 0.001, Cohen’s d = 0.725) and total brain volume (*p* < 0.001, Cohen’s d = 1.321).

### Mediation analyses

After evaluating whether mediation analyses conditions were met (i.e., all three paths were statistically significant), only 10 mediation models satisfied the conditions (Table 3). These models used the Fluid Intelligence, Paired Associate Learning, and Symbol Digit Substitution sub-samples. The first set of mediation models used the Fluid Intelligence task as the outcome (Fig. 1); it was observed that in women depression severity negatively influenced fluid intelligence score via the hippocampus (Indirect effect [IE]_PHQ-9 total_ = -0.001, *p* = 0.006; Proportion mediated [PM] = 2.9%). This was also found when investigating somatic symptom severity (IE_PHQ-9 somatic_ = -0.001, *p* = 0.008; PM = 3.9%) and cognitive/affective symptom severity (IE_PHQ-9 cognitive_ = -0.001, *p* = 0.026; PM = 0.023%).

**Table 3:** Direct and Indirect Effects of Depression Severity on Cognition as Mediated by Hippocampal Volumes

Measure	Women		Men	
	β or IE (95% CI)	*p-value*	β or IE (95% CI)	*p-value*
**Fluid Intelligence** (Women *n* = 9,300; Men *n* = 8,581)				
**PHQ-9 Depression Severity Total Score**				
Total effect	-0.021 (-0.033 to -0.01)	*p* < *0.001*	-0.023 (-0.037 to -0.010)	*p* = *0.002*
Direct effect	-0.021 (-0.032 to -0.01)	*p* < *0.001*	-0.023 (-0.037 to -0.010)	*p* = *0.002*
Indirect effect via hippocampus	-0.001 (-0.001 to 0.00)	*p* = *0.006*	–	*–*
Proportion mediated (%)	2.9%		–	
**PHQ-9 Somatic Symptoms Severity Score**				
Total effect	-0.026 (-0.048 to -0.010)	*p* = *0.020*	-0.027 (-0.052 to 0.000)	*p* = *0.040*
Direct effect	-0.025 (-0.047 to 0.000)	*p* = *0.020*	-0.028 (-0.053 to 0.000)	*p* = *0.040*
Indirect effect via hippocampus	-0.001 (-0.002 to 0.000)	*p* = *0.008*	–	*–*
Proportion mediated (%)	3.9%		–	
**PHQ-9 Cognitive/Affective Symptoms Severity Score**				
Total effect	-0.044 (-0.044 to -0.02)	*p* < *0.001*	-0.046 (-0.072 to -0.020)	*p* < *0.001*
Direct effect	-0.043 (-0.064 to -0.02)	*p* < *0.001*	-0.046 (-0.073 to -0.020)	*p* < *0.001*
Indirect effect via hippocampus	-0.001 (-0.002 to 0.00)	*p* = *0.026*	–	*–*
Proportion mediated (%)	0.023%		–	
**Paired Associate Learning** (Women *n* = 6,664; Men *n* = 5,970)				
**PHQ-9 Depression Severity Total Score**				
Total effect	-0.018 (-0.038 to 0.000)	*p* = *0.049*	-0.028 (-0.05 to -0.010)	*p* = *0.010*
Direct effect	-0.018 (-0.037 to 0.000)	*p* = *0.057*	-0.029 (-0.051 to -0.010)	*p* = *0.008*
Indirect effect via hippocampus	–	*–*	0.001 (0.0001 to 0.000)	*p* = *0.036*
Proportion mediated (%)	–		3.3%	
**PHQ-9 Somatic Symptoms Severity Score**				
Total effect	-0.035 (-0.035 to -0.069)	*p* = *0.037*	-0.040 (-0.040 to -0.079)	*p* = *0.041*
Direct effect	-0.034 (-0.034 to -0.068)	*p* = *0.043*	-0.041 (-0.041 to -0.081)	*p* = *0.032*
Indirect effect via hippocampus	-0.001 (-0.003 to 0.000)	*p* = *0.068*	0.002 (0.0001 to 0.000)	*p* = *0.026*
Proportion mediated (%)	NA		4.4%	
**PHQ-9 Cognitive/Affective Symptoms Severity Score**				
Total effect	-0.025 (-0.062 to 0.010)	*p* = *0.139*	-0.051 (-0.093 to -0.010)	*p* = *0.009*
Direct effect	-0.024 (-0.061 to 0.010)	*p* = *0.154*	-0.052 (-0.094 to -0.010)	*p* = *0.007*
Indirect effect via hippocampus	–	*–*	–	*–*
Proportion mediated (%)	–		–	
**Symbol Digit Substitution** (Women *n* = 6,602; Men *n* = 5,914)				
**PHQ-9 Depression Severity Total Score**				
Total effect	-0.063 (-0.097 to -0.03)	*p* < *0.001*	-0.111 (-0.150 to -0.08)	*p* < *0.001*
Direct effect	-0.062 (-0.095 to -0.03)	*p* < *0.001*	-0.112 (-0.151 to -0.08)	*p* < *0.001*
Indirect effect via hippocampus	-0.001 (-0.003 to 0.00)	*p* = *0.064*	0.002 (0.0002 to 0.00)	*p* = *0.020*
Proportion mediated (%)	NA		1.4%	
**PHQ-9 Somatic Symptoms Severity Score**				
Total effect	-0.113 (-0.173 to -0.050)	*p* < *0.001*	-0.164 (-0.232 to -0.10)	*p* < *0.001*
Direct effect	-0.110 (-0.171 to -0.050)	*p* < *0.001*	-0.167 (-0.236 to -0.10)	*p* < *0.001*
Indirect effect via hippocampus	-0.002 (-0.005 to 0.00)	*p* = *0.058*	0.003 (0.0004 to 0.01)	*p* = *0.020*
Proportion mediated (%)	NA		1.8%	
**PHQ-9 Cognitive/Affective Symptoms Severity Score**				
Total effect	-0.094 (-0.159 to -0.030)	*p* = *0.003*	-0.198 (-0.269 to -0.130)	*p* < *0.001*
Direct effect	-0.092 (-0.157 to -0.030)	*p* = *0.003*	-0.201 (-0.271 to -0.130)	*p* < *0.001*
Indirect effect via hippocampus	–	*–*	–	*–*
Proportion mediated (%)	–		–	

Displayed are the 10 of the regression-based mediation models that met all conditions for mediation analyses (e.g., all three paths were statistically significant) and their sex counterparts. Proportion mediated will be NA if mediation was not significant. If ‘–' the mediation analysis was not done due to the path conditions not being met. Indirect Effect (IE)

The second set of mediation models used the Paired Associate Learning task as the outcome. In men it was observed that the number of questions answered correctly was positively associated with depression severity via the hippocampus (IE_PHQ-9 total_ = 0.001, *p* = 0.036; PM = 3.3%). When investigating types of depression symptoms, only the somatic symptom severity mediation model was significant (IE_PHQ-9 somatic_ = 0.002, *p* = 0.026; PM = 4.4%).

The third set of mediation models used performance on the Symbol Digit Substitution task as the outcome. In men the hippocampus mediated the positive relationship between depression severity and number of correct symbol-digit matches (IE_PHQ-9 total_ = 0.002, *p* = 0.020; PM = 1.4%). Analyses evaluating types of depression symptoms only found a significant mediation for somatic symptom severity (IE_PHQ-9 somatic_ = 0.003, *p* = 0.020; PM = 1.8%).

Mediation analyses conditions were not met for the PHQ-9 score > 4 samples. After controlling for false discovery rate (FDR) using Benjamini–Hochberg procedure only the findings for the first set of mediation models using the Fluid Intelligence task remained significant in women (β_PHQ-9 total_ = -0.001, *p* = 0.019; β_PHQ-9 somatic_ = -0.001, *p* = 0.023; β_PHQ-9 cognitive_ = -0.001, *p* = 0.041). Findings in men for the Paired Associate Learning task and the Symbol Digit Substitution task mediation models did not survive FDR correction.

## Discussion

The goal of this study was to elucidate the sex-specific mechanisms by which depression confers risk of AD. This investigation found sex differences in the hippocampal-mediated relationship between depression severity and cognition in a sample of older adults without dementia. It was found that only in women hippocampal volumes mediated the relationship between depression severity and performance on the Fluid Intelligence task. It should be emphasized that it was found that less than 3% of the effect of depression severity on fluid intelligence is mediated by the hippocampus. Although there were initial findings in two cognitive tasks for men – the Paired Associate Learning and Symbol Digit Substitution – these results were not robust and did not survive FDR corrections.

The UK Biobank Fluid Intelligence task is a measure of fluid cognitive ability (i.e., verbal and numerical reasoning) (Fawns-Ritchie & Deary, 2020). Consistent with previous literature the present study showed hippocampal atrophy is related to fluid intelligence decline in older adults (Reuben et al., 2011). Evidence for sex differences in fluid intelligence are scare, however a few studies have found that minimal depression symptoms are negatively associated with fluid intelligence only in older men (Brevik et al., 2013; Rabbitt et al., 1995). These findings are not consistent with the present study in which fluid intelligence was only associated with depression in women but not in men. It is possible that this discrepancy can be attributed to the use of different depression measures. However, it is also possible that the sex differences found in this study, where men performed better on this task than women, could be the reason why women with greater depression severity scores seem to be at a greater disadvantage.

The exploratory analyses investigating sex differences in the association between depression symptom type and cognition found that in women both somatic and cognitive/affective symptom severity had a negative relationship with Fluid Intelligence performance. These findings suggest depression severity results in specific cognitive vulnerabilities for women, and that there might be distinct pathways by which somatic and cognitive/affective depression symptoms manifest and influence cognition. As is consistently found in studies examining sex differences in depression, women in this study had greater depression severity, as well as somatic and cognitive/affective symptom severity (Silverstein, 2002). However, it is important to note that lower depression severity scores in men could perhaps be explained by men typically under reporting symptoms of depression when compared to women. As has been highlighted by others it is possible that depression in men might manifest through symptoms that are not captured by current depression measures (i.e., engagement in risk-taking behaviors or substance use) (Alexopoulos, 2005; Cavanagh et al., 2017; Khan et al., 2002). These findings suggest a need for studies that focus on better understanding sex differences in how depression manifests in older adults.

### Limitations

This study focused on the hippocampus a priori due to its functional relevance in the neurotoxicity hypothesis and its relevance to memory and AD, but future studies will examine other brain regions implicated in depression. It should be noted that this study controlled for total brain volume (TBV) instead of intracranial brain volume since TBV was provided within the UK Biobank’s IDPs. It is also important to note that there could be potential hemispheric lateralization effects that need to be explored in future studies. In addition to the homogenous and highly educated UK Biobank sample, there are inherent limitations in the current study that could impact the generalizability of its findings. Firstly, since the aim of the study was to explore the impact of depression, in the form of symptom subtypes and severity, the measure available to assess this within the UK Biobank was limited to the PHQ-9. Additionally, the PHQ-9 was completed via the online Mental Health Questionnaire which was administered outside of the timeframe and environment in which the cognitive measures and the MRI were acquired. To account for this the authors included time between key study variables as a covariate in the analyses. Secondly, the cognitive testing was brief, unsupervised, and administered on a touchscreen which could have introduced bias against older adults that are less technologically savvy. Additionally, due to missing cognitive data this study had different sample sizes for each cognitive domain being evaluated. Thirdly, in connection to the last point, the authors were not able to ascertain the reasons for missing data within the variables used in this study. Therefore, it could be that the participants analyzed in this study are not representative of the entire UK Biobank cohort. Lastly, the unknown presence of confounding variables provides another limitation. At the time the PHQ-9 was completed participants were not asked about current pharmacological or non-pharmacological treatments for mental illnesses.

## Conclusions

This study offers a first insight into the sex-specific mechanisms that underlie the relationship between depression, cognition, and the hippocampus in older adults without dementia. The hippocampus was found to partially mediate the association between depression severity and cognitive performance within specific domains differently by sex. These results suggest depression could confer risk of AD differently in men and women by contributing to the vulnerability of specific cognitive domains. Additionally, these findings allude to other biological factors (e.g., APOE-ε4, cortisol, inflammation, BDNF) being potential mediators for the relationship between depression and cognition that is not directly mediated by the hippocampus. Since depression can manifest at any stage of life and episodes can span weeks to months, it is important to look beyond depression experienced at one time point. Future studies should explore lifetime history of depression and an individual’s length and number of depressive periods. Studies should also focus on investigating sex differences in how depression manifests in older adults to be able to harness the prognostic power of depression for AD. This knowledge along with the findings from this study could aid in the development of targeted dementia prevention strategies and treatments for men and women.

## Supplementary Information

Below is the link to the electronic supplementary material.

Supplementary file1 (JPG 1.64 MB)

Supplementary file2 (DOCX 17.8 KB)

## Data Availability

The dataset used for the current study is not publicly available as there is a data access application required by the UK Biobank. Information on how to obtain the data can be found via the UK Biobank’s website (https://www.ukbiobank.ac.uk/enable-your-research/apply-for-access). Details on how to reproduce the dataset for the current study’s analysis is available from the corresponding author upon request.

## Code availability

Available upon request.
