# Supplementary figures and images for: Sex influences whether hippocampal volumes mediate the relationship between depression and cognition in older adults without dementia: A UK Biobank study
Source: Brain Imaging Behav. 2024 Oct 11;19(1):12–22. doi: 10.1007/s11682-024-00930-6 (PMC11846722; doi:10.1007/s11682-024-00930-6)

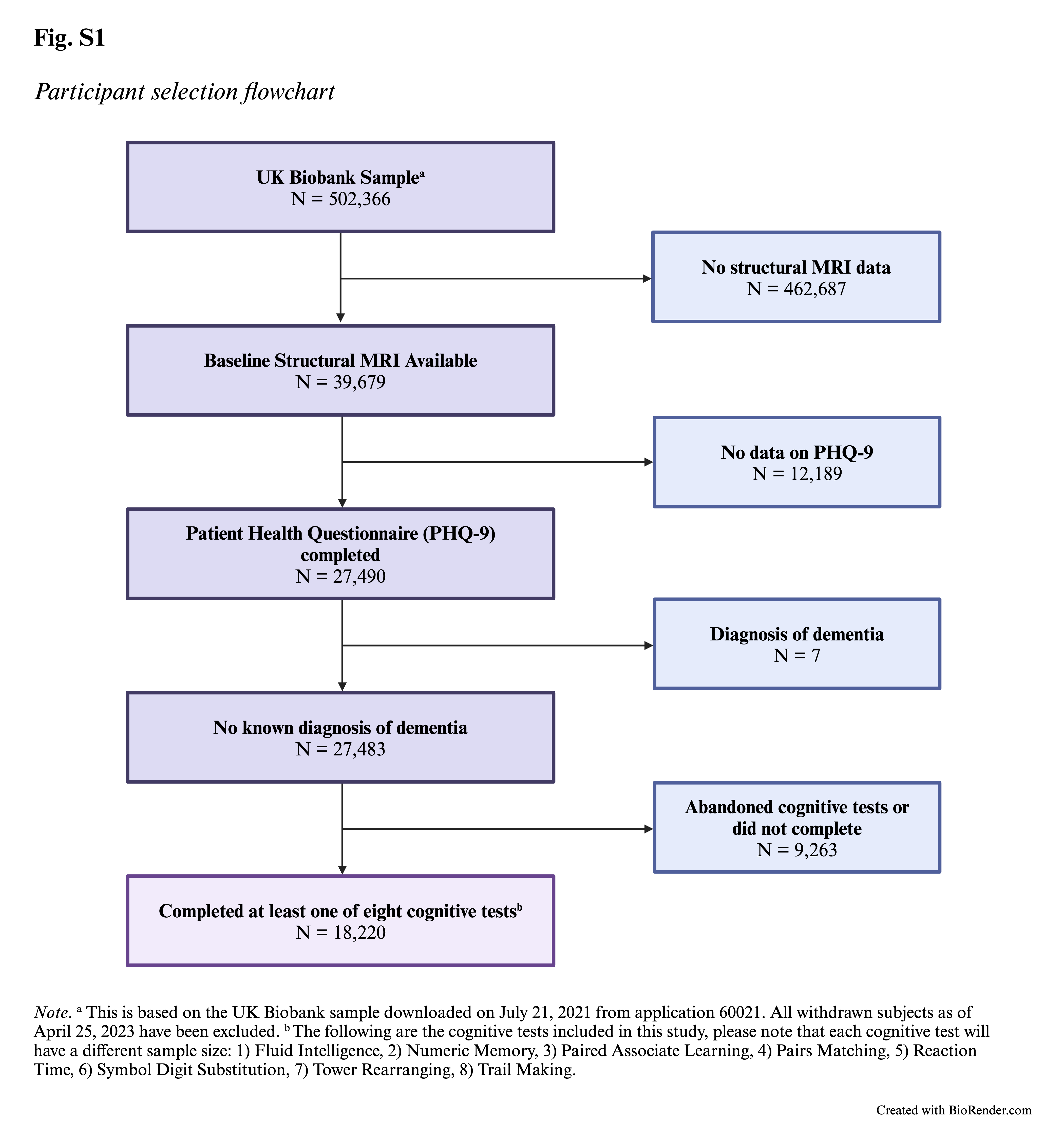

Supplement: Supplementary file 1 — Supplementary file1 (JPG 1.64 MB) [file 11682_2024_930_MOESM1_ESM.jpg]
