# Supplementary material for: Sex influences whether hippocampal volumes mediate the relationship between depression and cognition in older adults without dementia: A UK Biobank study
Source: Brain Imaging Behav. 2024 Oct 11;19(1):12–22. doi: 10.1007/s11682-024-00930-6 (PMC11846722; doi:10.1007/s11682-024-00930-6)
Supplement: Supplementary file 2 — Supplementary file2 (DOCX 17.8 KB) [file 11682_2024_930_MOESM2_ESM.docx]

| **Table S1**  *PHQ-9 score > 4 Samples for Sensitivity Analyses* | | | | | |  |  |  |  |  |  |  |
| --- | --- | --- | --- | --- | --- | --- | --- | --- | --- | --- | --- | --- |
|  | **Fluid Intelligence** | | | | **Paired Associated Learning** | | | | **Symbol Digit Substitution** | | | |
|  | **Women (n=1,647)** | **Men (n=1,118)** | **Effect Size** | ***p-value*** | **Women (n=1,208)** | **Men (n=810)** | **Effect Size** | ***p-value*** | **Women (n=1,192)** | **Men (n=796)** | **Effect Size** | ***p-value*** |
| **PHQ-9 Depression Severity Total Score** | 8.24 ± 3.86 | 8.02 ± 3.77 | 0.06 | *0.130* | 8.28 ± 3.89 | 8.14 ± 3.93 | 0.04 | *0.440* | 8.29 ± 3.90 | 8.14 ± 3.96 | 0.04 | *0.390* |
| **PHQ-9 Cognitive/Affective Symptoms Severity Score** | 3.78 ± 2.59 | 3.90 ± 2.65 | 0.04 | *0.270* | 3.80 ± 2.60 | 3.89 ± 2.67 | 0.03 | *0.460* | 3.82 ± 2.60 | 3.90 ± 2.68 | 0.03 | *0.500* |
| **PHQ-9 Somatic Symptoms Severity Score** | 4.46 ± 2.08 | 4.12 ± 2.08 | 0.16 | *<0.001* | 4.48 ± 2.11 | 4.25 ± 2.16 | 0.11 | *0.010* | 4.48 ± 2.11 | 4.24 ± 2.16 | 0.11 | *0.020* |
| **PHQ-9 Diagnosis** |  |  | 0.05 | *0.060* |  |  | 0.05 | *0.170* |  |  | 0.07 | *0.140* |
| *Mild depression* | 74.40% | 78.80% |  |  | 74.00% | 77.80% |  |  | 73.70% | 77.80% |  |  |
| *Moderate depression* | 17.60% | 14.60% |  |  | 17.40% | 14.90% |  |  | 17.60% | 14.90% |  |  |
| *Moderately severe depression* | 5.30% | 4.10% |  |  | 5.90% | 4.30% |  |  | 5.90% | 4.30% |  |  |
| *Severe depression* | 2.70% | 2.50% |  |  | 2.70% | 3.00% |  |  | 2.80% | 3.00% |  |  |
